# ‘There is trauma all round’: A qualitative study of health workers’ experiences of caring for parents after stillbirth in Kenya and Uganda

**DOI:** 10.1016/j.wombi.2022.02.012

**Published:** 2023-02

**Authors:** Tracey A. Mills, Elizabeth Ayebare, Jonan Mweteise, Allen Nabisere, Raheli Mukhwana, Anne Nendela, Grace Omoni, Sabina Wakasiaka, Tina Lavender

**Affiliations:** a Centre for Childbirth, Women’s and Newborn Health, Department of International Public Health, Liverpool School of Tropical Medicine, Liverpool, UK; b Department of Nursing, College of Health Sciences, Makerere University, Kampala, Uganda; c Lugina Africa Midwives’ Research Network, Kampala, Uganda; d Lugina Africa Midwives’ Research Network, Nairobi, Kenya; e School of Nursing Sciences, University of Nairobi, Nairobi, Kenya

**Keywords:** Stillbirth, Bereavement care, Midwives, Global health

## Abstract

**Background:**

Stillbirth is a traumatic life-event for parents. Compassionate care from health workers supports grief and adjustment, alleviating psychological distress and minimising serious adverse health and social consequences. Bereavement support in facilities in LMICs, including in sub-Saharan Africa, often fails to meet parents’ needs. However, very few studies have explored health worker’s experiences in these settings.

**Aim:**

To explore the lived experiences of midwives, doctors and others, caring for women after stillbirth in Kenya and Uganda.

**Methods:**

Qualitative, guided by Heideggerian phenomenology. Sixty-one health workers, including nurse-midwives (N = 37), midwives (N = 12) and doctors (N = 10), working in five facilities in Kenya and Uganda, were interviewed. Data were analysed following Van Manen’s reflexive approach.

**Results:**

Three main themes summarised participants’ experiences: ‘*In the mud and you learn to swim in it*’ reflected a perceived of lack of preparation; skills were gained through experience and often without adequate support. The emotional and psychological impacts including sadness, frustration, guilt and shame were summarised in *‘It’s bad, it’s a sad experience’*. Deficiencies in organisational culture and support, which entrenched blame, fear and negative behaviours were encapsulated in *Nobody asks ‘how are you doing?’*.

**Conclusion:**

Health workers in Kenya and Uganda were deeply sensitive to the impacts of stillbirth for women and families, and often profoundly and personally affected. Care and psychological support were acknowledged as often inadequate. Interventions to support improved bereavement care in sub-Saharan Africa need to target increasing health worker knowledge and awareness and also embed supportive organisational cultures and processes.

**Statement of significance**

**Problem?**

In LMICs, bereavement support for parents after stillbirth in health facilities is often inadequate

**What is already known?**

Compassionate care from health workers supports grief and adjustment, minimising adverse health and social outcomes for parents. The experiences of health workers in sub-Saharan Africa, which bears a high burden of stillbirth, are poorly understood.

**What this paper adds?**

Health workers in Kenya and Uganda were profoundly affected, and often frustrated by their inability to support women and families after stillbirth. Specific preparation and education, especially in communication skills were lacking. Institutional cultures emphasising blame and fear around adverse outcomes negatively impacted behaviours and were a barrier to compassionate care.

## Introduction

In 2020 the WHO/UNICEF ‘Renewed call for collective action’ [1] re-invigorated the global commitment to tackling stillbirth as a neglected maternal and newborn health issue. The overwhelming burden of two million annual stillbirths falls on LMICs, with sub–Saharan Africa accounting for more than half of all deaths. Kenya and Uganda report stillbirth rates around eight times higher than HICs [2]. Beyond these shocking numbers, every death of a baby before or during birth is a tragedy for parents and families. Grief is long-lasting and often not socially validated, leading to isolation. Stillbirth is highly stigmatised in many communities [3]. Mothers are reported to experience substantial adverse impacts. An estimated 4.2 million women are living with depression related to their baby’s death and relationship breakdown is also common [4]. In LMICs, debilitating physical morbidity related to traumatic birth, including obstetric fistula may also amplify these effects. Stillbirth is also associated with wider negative social sequelae including increased health care costs and lost economic productivity [5].

Alongside stillbirth prevention *‘ensuring appropriate bereavement support for parents and families when a baby dies’* was recognised as a key WHO/UNICEF priority [1]. Most births in LMICs, including Kenya and Uganda, now take place in health facilities. Therefore, health workers have direct and intimate contacts with women and families during childbirth, including when a baby dies. Compassionate care and support in the hours and days after stillbirth is recognised as critical in minimising trauma and protecting against poor psychological outcomes [6]. However, accumulating evidence suggests that many parents in LMICs, notably sub-Saharan Africa, do not receive appropriate care in health facilities after stillbirth [7], [8], [9]. Insensitive communication, deficiencies in information and emotional support are reported, reflecting negative experiences also described in HICs. Whilst parent’s experiences have been explored in some LMIC settings, there is much less evidence surrounding health workers’ views and perceptions surrounding practice [8], which are critical to improving care in these settings. This study aimed to explore lived experiences of health workers in providing care to bereaved women and families in facilities in Kenya and Uganda.

## Methodology and methods

### Study design

This study, part of a research programme exploring bereavement support in sub–Saharan Africa, was guided by Heideggerian phenomenology [10], and sought to capture the experiences and perspectives of a variety of maternity care providers including nurse-midwives, midwives and doctors. Heidegger rejects suspension of researcher preconceptions but mandates reflexivity throughout the process. Eight authors were midwives or nurse-midwives (two were UK-based academic midwives) and one was a public health nurse. All the authors have had personal experience of caring for parents following perinatal death, seven had direct experience of providing maternity care in Kenya or Uganda.

### Patient and public involvement

In both countries, stakeholder groups of academics, clinicians and policymakers and community engagement and involvement (CEI) groups of parents who had previously experienced stillbirth supported the research, from design to interpretation and dissemination. Support and training for CEI groups were provided through the research programme partnership [11].

### Ethics

Research governance approvals were confirmed with the appropriate institutional ethics committees in UK, Kenya and Uganda including the Uganda National Council for Science and Technology. Administrative clearance was also obtained from the included facilities, before study commencement and written informed consent was obtained from all participants, including for use of anonymised verbatim quotes.

### Setting and participants

A purposive sample of health workers, employed at five urban, peri-urban (areas of transition between urban and rural environments) and semi-rural maternity facilities in Nairobi and Western Kenya (three facilities), Kampala and Central Uganda (two facilities) were recruited. Eligibility criteria included regularly providing care for women and families after the death of a baby. Prior to recruitment, workshops were held in the included facilities to inform staff and information leaflets were distributed with research team contact details. Those interested in participating were invited to contact the research team directly, for further information. Interviews were arranged at a mutually convenient time and location, often a private room at the facility. The sample size was estimated at 10-15 participants per site, aimed to provide data adequacy [12]. Although data saturation is not a pre-requisite for phenomenology, we consider that this was achieved as no new themes emerged at the conclusion of analysis.

### Data collection

One-to-one interviews were conducted by experienced research assistants with a midwifery or nursing background. Investigator-designed questionnaires were used to collect demographic and practice data. A topic guide, based on the literature and discussions with stakeholder and CEI groups, was used. A broad opening question was used to invite participants to share their practice experiences with women and families after stillbirth, they were encouraged to respond without interruption. Minimal prompts were used to clarify meaning and boost depth where required. Interviews were conducted in English, which is the language of clinical practice in both countries, digitally audio-recorded and transcribed verbatim. Participants chose a pseudonym to protect their identity. A verbal summary of the main points was provided to the participant by the interviewer at the end of each interview, to confirm the accuracy of interpretation. Contemporaneous field notes captured nuances and a reflexive diary entry, made after completion documented interview dynamics, learning points and emerging themes. Recognising the sensitivity of the topic, a study-specific distress policy, outlining the immediate and follow-up actions to be taken in the event of participant distress during an interview was available. This included a process for referral and signposting to local counselling services for ongoing support, if required and the participant agreed. As experienced health workers, the interviewers had the requisite skills to support participants appropriately during data collection. No participant requested or required referral for ongoing support.

### Analysis

Transformation of individual lived-experiences into textual expression of their meaning was guided by van Manen’s reflexive approach [13], using themes as structures of meaning. This involved a three-stage process; firstly, a ‘wholistic [sic] approach’ considering the narratives in their entirety by reading and re-reading transcripts, achieving familiarisation. Secondly, a selective approach, where sections of interest were highlighted with memos attached documenting reasons. Next, a ‘detailed approach’ involved text being considered line-by-line and sentences placed into clusters, according to commonalities. The initial analysis involved two researchers in each country and one UK-based researcher. Noting many commonalities in the initial analyses across both countries, subthemes and themes were generated with input from the whole research team at a series of meetings. This involved amalgamation of clusters, frequently returning to individual texts and field notes for confirmation following the ‘hermeneutic circle’, a key concept of Heidegger’s approach [14]. Summaries of emerging concepts were presented to the local stakeholder and CEI groups and feedback was shared, to confirm the final interpretation.

## Findings

Interviews were conducted with 61 health workers (Kenya N=41, Uganda N=20), including 37 nurse-midwives, 12 midwives and 10 doctors, all currently practicing in the included facilities. Participant characteristics are presented in Table 1. Three main themes summarised interpretation of participants’ experiences of care for women and families after the death of a baby. Except where stated, findings were common across both countries and professional groups, the theme titles include verbatim quotes. Theme 1 ‘*In the mud and you learn to swim in it*’ summarises practice experiences and development of skills in three subthemes. These included communication with enabling factors described in subtheme ‘*Try to walk in their shoes’* and barriers and challenges encountered in ‘*I don’t have the right words’*, experiences surrounding facilitating seeing the baby after birth are summarised in *‘Contacts with the baby’*. Theme 2 *‘It’s bad, it’s a sad experience’* illustrates the emotional impacts of stillbirth including two subthemes *‘Guilt blame and fear’* and *‘What did I do wrong, what didn’t I do?’* reflecting assignment of blame, dealing with families and complaints and litigation. Issues surrounding organisational support and culture are addressed in Theme 3: ‘*Nobody asks ‘how are you doing?’.*

**Table 1:** Participant characteristics (N=61).

Country		Kenya (n=41)	Uganda (n=20)	Total (n=61)
**Gender**	Female	33 (80%)	13 (65%)	46 (75%)
	Male	8 (20%)	7 (35%)	15 (25%)
**Role/Job title**	Doctor	3 (7%)	7 (35%)	10 (16%)
	Midwife	2 (5%)	10 (50%)	12 (20%)
	Nurse-Midwife	34 (83%)	3 (15%)	37 (61%)
	Other	2* (5%)	0	2 (3%)
**Highest level of education**	Certificate/Diploma	34 (83%)	10 (50%)	44 (72%)
	Degree	5 (12%)	8 (40%)	13 (20%)
	Postgraduate	2 (5%)	2 (10%)	4 (3%)
**Post-qualification experience (years; median and range)**		16 (1-30)	12 (1-23)	15 (1-30)
**Personal or family experience of perinatal death**		16 (39%)	8(40%)	24 (39%)
**Bereavement care education (pre or post registration [reg])**	No	40 (97%)	18 (90%)	58 (95%)
	Yes	1 (pre-reg)	2 (pre-reg)	3 (5%)

All data are n (% of country or total) unless stated. *Other participants included 1 hospital social worker, 1 reproductive health counsellor.

### Theme 1: ‘In the mud and you learn to swim in it’

Baby deaths were very common in all the included facilities and participants encountered bereaved women and families very regularly. Both doctors and midwives viewed bereavement care as amongst the most demanding aspects of their work. Very few recalled any specific pre or in-service (before or after qualification) education and guidance, and protocols were not available in the facilities. Practice was largely based on experience, but this was often gained without support giving rise to anxieties about adequacy and quality:

*‘Remember they came happy, fully prepared maybe even named the baby then (silence….1 min) then I say what? That I did not do my best or that I don’t know what happened? Besides, we have never received any training on care and support-one just finds herself in the “Mud” and you learn to swim in it.’ (Mary, Doctor, Kenya)*

### ‘Try to walk in their shoes’

Communication with bereaved parents, from sharing the news of the baby’s death to providing ongoing information and explanations was a particular challenge, even for the most experienced. Amongst influences on their practice, health workers spoke of deep empathy for women and related how they tried to ‘put myself in their shoes’ when caring for women. Some also drew on personal or family experiences of the death of a baby when approaching conversations. A few recalled support from senior colleagues as helpful early in their careers, observation and reflection on peer practice was also considered beneficial in developing skills:

*‘I went to the office where we used to stay so I told the sister in-charge, “you are the one going to explain because I don’t know where to start.” So she went to explain to the mother about it, the mother wasn’t so negative, I think she understood. The sister in-charge had that experience of explaining things. (John, Nurse-Midwife, Kenya)*

*‘I don’t have the right words’*

Sharing bad news was always viewed with trepidation and many health workers expressed specific concerns around what to say, when and how. The circumstances of the baby’s death affected the degree of worry and apprehension experienced. Where stillbirth occurred before labour, was preceded by a complication (e.g., reduced fetal activity or vaginal bleeding) and could be confirmed (e.g., by ultrasound scan), it was felt to be less stressful by health workers because women were likely ‘to expect bad news’. Telling a woman that her baby had died during labour or after emergency caesarean was much more challenging:

*“the mother goes to the delivery room feeling the foetal movement and the foetal heart can be felt by the health care provider only for the foetal heart rate stops abruptly in the delivery room. This is challenging as the mother is expecting to give birth to live baby only to get a still birth. For a health worker facing the mother there in the delivery bed with her dead baby in your hands it is most difficult experience.” (Cecilia, Nurse-Midwife, Kenya)*

Uncertainty around outcomes, often precipitated by resource shortages, added to stress around communication for health workers during labour. Stillbirth was sometimes suspected, for example when the fetal heart could not be auscultated clearly, but often could not be confirmed due to of lack of access to ultrasound. Also, caesarean section for identified fetal compromise was often delayed because of lack of theatre capacity. Some health workers, shared information in these situations to try to ‘prepare’ women.

*“If we get fetal distress for example and we don’t have theatre space, and you suspect that you may get a fresh stillbirth. So we always try to make sure we document and we inform these mothers that “you know, your baby is tired but we don’t have space in the theatre right now, but we are going to try as much as we can.’” (Darwin, Doctor, Uganda)*

However, often intrapartum stillbirth was unanticipated and sometimes was only apparent at birth. In these stressful circumstances, midwives spoke of the need to react rapidly and provide appropriate clinical care (e.g., initiate resuscitation attempts) but also to inform and support women and families. Responding to multiple demands, often without colleagues to assist, was challenging. After vaginal birth, women invariably realised something was wrong when the baby did not cry and became extremely distressed. Where resuscitation was attempted, babies often needed to be removed from the birthing area to access limited equipment. This meant women were left alone as labour companions were also rarely present, and communication inevitably delayed. In most cases the health worker conducting the birth informed the mother (and family, if present) although doctors sometimes delegated this task to the midwife, citing pressure of other work. Shock and denial were common responses; many health workers recalled women and families accusing them of ‘swapping’ the live baby for a stillborn one.

*Mostly verbally, they are harsh to the midwives, asking the midwives, “what have you done to the baby?” My baby was okay? I was feeling the foetal movement, what have you done to the baby maybe during delivery?” (Wendy, Nurse- Midwife Kenya)*

When confronted with extreme emotions, participants felt they lacked ‘the right words’ to comfort women and brief expressions of regret and condolences offered were insufficient. Midwives’ feelings of inadequacy were compounded by being unable to give time to bereaved women after the birth, as they usually cared for several women simultaneously:

*‘So you just leave her there crying. You have no time to come back again to console her, to have time with her for her to tell you what she thinks about. We no longer ask mothers what they are thinking about because we don’t have time for that.’* (Agaba, Nurse-Midwife, Uganda)

Sharing news of a baby’s death after caesarean section presented further dilemmas, there was a need to balance timely information with the woman’s capacity to receive this. General anaesthesia and ongoing maternal complications might necessitate delay. Health workers also encountered pressure from families to withhold information particularly if the woman was unwell. This might mean she was not told of the death for several hours or days after birth. Some health workers were deeply uncomfortable with such requests and risked conflict with families to uphold women’s interests, like Ruth:

*‘She will cry but we have nothing to do we have to tell her the truth because if we don’t, some can even spend like three days being lied to. ‘The baby is on oxygen’. when the baby has already passed away and it has already been buried. So we tell them. Personally, I don’t like keeping a mother waiting for so long thinking her baby is somewhere when it is already dead so I will tell them.’* (Ruth, Midwife, Uganda).

### Contact with the baby

In both countries, women were actively encouraged to see their stillborn baby after birth. This was considered important to confirm that there were no signs of life and the baby’s sex, therefore sometimes only genitalia were exposed. In many facilities women, partners or relatives were required to confirm this in writing. In addition to promoting ‘acceptance’ that the baby had died, this was believed to protect staff against accusations of ‘swapping’ and associated complaints. No participant mentioned discussing seeing the baby with women in advance, although some acknowledged cultural prohibitions against contact with the dead which made some women reluctant. Rapid burial was a cultural norm in both countries and if a mother was ill or unconscious, staff often had to persuade relatives to delay so they could facilitate viewing.

*‘She has to find out what was the sex of the baby. She has to know about that. So we hand over the body to the people concerned and we always allow them to take the baby in case the mother is conscious. To those of caesar [caesarean] we have to wait for the mother to become conscious, she takes a look on the baby and the baby is taken for burial.’ (Josephine, Midwife, Uganda)*

### Theme 2: ‘It’s bad, it’s a sad experience’

Notwithstanding relentless exposure to poor outcomes in both Kenya and Uganda, caring for women and families after the death of a baby evoked powerful emotional reactions amongst many participants.

*I feel heartbroken… I carry that woman as myself. What is she going to take home? It breaks my heart completely (Juliet, Nurse-Midwife, Kenya)*

Caring for bereaved parents often reawakened personal or family experiences and difficult memories. Sarah, who had a stillbirth herself a few years earlier related this poignantly:

‘*it felt like I was the one going through the stillbirth experience, you could feel it like it’s you. It was so painful. By the way I also cried with them. Yes, my tears could not hold.’ (Sarah Nurse-Midwife, Kenya)*

Some individual circumstances evoked particular sadness, for example when a woman of advanced age having her first baby, history of infertility or repeated previous pregnancy losses had a stillbirth. Beyond empathy for her loss and grief, they were highly conscious of the potential family and social consequences for women of the loss of a ‘precious baby’, including isolation, stigma, abandonment, and divorce:

*‘And one time a mother who was now having, I think it was either a third or second [stillbirth], it was difficult. She was saying, ‘’now I am out of the marriage. They are going to chase me out.’ (Betty, Nurse-Midwife, Uganda).*

### Guilt, blame and fear

In both countries, many stillbirths were viewed as preventable and health workers expressed considerable frustration at the persistence of high numbers. When death occurred before admission, there was a tendency for health workers to blame women and families for delaying seeking skilled care. In Uganda, health workers particularly focused on widespread preference for traditional birth attendants or using herbal medicines:

*But what I have observed, we tend to put the blame onto them like it’s them who caused the death. If she took herbal medicine even if it was a drop which didn’t have an effect you will put the blame (Ritah, Midwife, Uganda)*.

*‘What did I do wrong, what didn’t I do?’*

The death of a baby during labour provoked a very different response, in these cases health workers often ruminated over events in depth. They frequently assumed personal responsibility, for example declaring that *‘we have lost this baby’*, even where health system deficiencies had obviously contributed. As facility birth was strongly advocated to improve outcomes, stillbirth was felt to be a betrayal of women’s trust in health workers and the system. Such guilt had detrimental impacts on morale with several participants expressing thoughts of leaving their profession.

*I usually ask myself a question “If only I could have had this mother gone to theatre this could not have happened” so it keeps on clicking in your mind. So I become down and you feel demoralized. We feel very sorry and it demoralizes, you regret that only one theatre was operational and if only had another theatre, we could have saved that baby. I feel demoralized to an extent that I feel like quitting the profession. (Mary, Midwife, Kenya).*

Many doctors and midwives had experienced angry, abusive and occasionally violent reactions from women, partners and family members. Mistrust, lack of prior contact, and relatives’ shock and distress led to tense, difficult situations. In the aftermath, health workers often felt traumatised and sometimes fearful for their physical safety. Anne, a labour ward midwife, recalled a particularly unpleasant incident when she was pregnant:

*When the husband came, I tried to talk to [him] about what has happened and he was blaming the hospital…blaming everybody…. I was pregnant… eight months and he was just saying ‘I wish it was your baby who died.’ Yes, it was very bad…I cried up to the third day. (Anne, Midwife, Kenya).*

In both countries, there was an escalating fear of complaints and litigation. Midwives, who conducted most of the births, often recalled experiencing accusations of negligence or poor practice from families. In Kenya, several related experiences of disgruntled relatives contacting the police or local media with complaints, for example unfounded accusations of exchanging/selling live babies for stillborn babies. A particularly troubling incident involved a stillborn baby misplaced in the facility mortuary when relatives came to view, a complaint to the police led to the midwife being held in a police cell until the error was resolved:

*I was very fearful for the first month and even to conduct deliveries, because I was wondering what if there is a Still-Birth or anything like that…what will happen? You know once beaten twice shy. So I was very,… very worried and I took time to calm down. Even now I have never recovered, it still comes in my mind and it is still very fresh in my mind. (Alice, Midwife, Kenya)*

Negative experiences, anecdotes and media reports were often felt to have undesirable influences on practice. Participants expressed reluctance to discuss causes of stillbirth with parents, for fear of implicating themselves or colleagues, others tried to avoid any conversations with bereaved mothers or families. In some facilities partner and family visiting was discouraged for fear of abuse or complaints. Women’s and families contacts with their stillborn baby immediately after birth were viewed by health workers as primarily to confirm the outcome, rather than to support grieving. The increasing insistence on women (and sometimes relatives) confirming death in writing immediately after birth and before release to the mortuary or burial raised some disquiet. Some midwives worried that the growing emphasis on self/institutional protection came at the expense of woman-centred care:

*“to me it feels a bit rude, because you wanted to be done there and then after birth before you have taken away the dead body. If it was like you first be with this mother like showing that empathy to her you would not be like that…. you [should] come back maybe you clean up the mother, make her comfortable, keep on checking on her, the bleeding and generally how she is there but immediately after birth you want to first show them their stillbirth and also consent for it. (Serena, Midwife, Uganda)*

### Theme 3: ‘Nobody asks ‘how are you doing?’’

Participants recognised the importance of their role in supporting women and families and that giving good quality bereavement care could also be satisfying for staff involved:

*‘Actually caring for them is good because there is a way these mothers feel we are part of them, we take care of them, they feel we are concerned, they feel that at least they are cared for so I really feel good to care for them.’ (Josephine, Midwife Uganda)*

In addition to addressing resource limitations and environmental barriers, a need for better preparation for providing bereavement support and communicating difficult news was identified. Many thought this should be included in pre-service education. Midwives and nurses were perceived as generally more ‘skilled’ in psychological support/communication, several doctors related valuable interprofessional learning experiences which had improved their skills in caring for bereaved families:

*‘You will find the matron in-charge, the senior nurse, my experience is that they have more exposure, and they normally take charge. And most especially young doctors, they have an opportunity and most of us we have learned from the nursing fraternity in terms of how to break the bad news, how to handle such situation.’ (Mike, Obstetrician, Kenya).*

However, participants also strongly believed education alone would not be sufficient to improve bereavement care, organisations needed also to recognise impacts and develop more effective support for staff. In some facilities, any open discussion of stillbirth was difficult and midwives in both countries described reluctance to be publicly associated with poor outcomes, fearful of criticism of their practice and damage to professional reputations within the workplace. One midwife spoke of the research interview being the first time anyone had asked her how she felt:

*Eh! That’s never talked about, it’s like taboo…. All these years everybody in maternity i.e. the midwives, obstetricians, managers and students never talk about it. If they do everybody talks in low tones as if it’s sinful to discuss the SB. (Sally, Nurse-Midwife, Kenya).*

Ineffective teamwork was also highlighted, midwives recalled feeling abandoned by doctors who left immediately after completing clinical tasks, without sharing information directly with women or families. Some of the less experienced doctors and midwives admitted passing responsibility for communication and care to others wherever they could. Communication between health workers around outcomes was also inadequate. No participant was aware of any specific support or counselling available to them. In Kenya, several midwives recounted personal experiences of unsupportive, censorious, and even abusive responses from managers after stillbirths, sometimes in public:

*‘So my in-charge was very hash on me telling me that I should not have allowed the doctor to put up the syntocinon…[she] told me “you are a murderer”. I went to my house and locked myself in the bedroom and cried. I didn’t have anywhere to go for counselling; If they have a system in place I would have gone back and be counselled. Because even going back to work you still have to take care of those mothers…. you have been traumatized…you feel bad. (Alice, Midwife, Kenya)*

## Discussion

This study explored the lived-experiences of health workers, including midwives, nurse-midwives and doctors, of caring for women and families after stillbirth across health facilities serving urban and rural communities in Kenya and Uganda. Despite relentless exposure to poor outcomes in Kenya and Uganda, health workers were profoundly personally affected by baby death. They were highly empathetic to women’s and families’ grief and genuinely recognised the importance of appropriate support. However, a multiplicity of internal and situational influences negatively impacted their capabilities to provide care as they would have wished. Knowledge and skills deficits, particularly lack of confidence in communication were a common concern. Resource shortages, notably low staff numbers, were also prominent, but negative practice cultures and lack of organisational support also acted as a barrier to improvement.

Health workers in this study described a range of emotions including feelings of failure, frustration and guilt, in common with responses by others providing care after stillbirth in other settings [15], [16]. These negative feelings were exacerbated by the absence of specific perinatal bereavement education and development. Perceived inadequacy led to some health workers to actively avoid bereaved women and families, ‘distancing’ as a form of self-protection has been reported in several previous studies in HIC [17]. Some staff also expressed desire to change their careers. Emotional exhaustion and withdrawal are associated with increased risk of ‘burn out’ syndrome [18]. Amongst health workers, midwives may be at particular risk and there is increasing recognition of ‘burn-out’ as a significant barrier to quality maternity care in LMICs [19], [20]. Personal or close family experience of the death of a baby was also relatively common amongst participants in this study. There have been few studies surrounding impacts of personal loss experiences on midwives’ and other health workers’ practice, but there is potential for increased risk of stress and trauma as a result [21], [22].

Increased knowledge and participation in educational experiences are associated with more positive attitudes and higher self-efficacy in midwives, factors associated with improved performance [23]. Skills for relational as opposed to technical care, especially for communicating difficult news were identified as a particular gap. Experiential approaches, allowing active learning in a safe environment, and incorporating reflection on practice experiences have established value in this area [24]. Resources and release of staff from clinical duties for training is a challenge in many LMIC facilities. Research around communication education for difficult conversations in health care in LMICs is limited, but a one-day workshop including simulation and role play decreased anxiety and led to sustained increase in self-reported knowledge, confidence and practice skills in US paediatric critical care staff [25]. Furthermore, this study and others demonstrated the added value of interprofessional learning, bringing health workers of different disciplines and levels of experience which might better approximate to the actual environment of practice and bring benefits in increasing mutual understanding, respect and breaking down hierarchies [8], [25], [26]. In the current study, doctors frequently acknowledged the value of observing midwives’ practice for developing their own communication skills.

Whilst the lack of bereavement education and training for staff in LMICs has previously been acknowledged [8], participants in this study also consistently highlighted the influence of practice culture and institutional factors on care. Some positive role models and examples of good support for junior staff were identified, however, many health workers expressed considerable anxiety, guilt, and fear of repercussions, particularly related to intrapartum stillbirths. Abuse from families, threats of complaints and litigation were perceived to be increasing, similar experiences have recently been reported amongst health workers caring for women after stillbirth in Lao [27]. Blame was a recurrent theme; communication and care delivery were often undesirably affected by fear of being held personally responsible for poor outcomes. Some health workers faced open criticism and abuse from managers and colleagues when error was perceived. Emergence of a ‘blame culture’ in maternity facilities in LMICs is increasingly reported [28], evolving from rule-orientated management styles which focus on assigning responsibility to individuals for system-level failures. Fear and distrust amongst health workers, characteristic of this culture, act to supress openness, practice learning and innovation and result in increasing errors and poor-quality health care [29]. For example, the existence of blame culture has recently been acknowledged as a barrier to perinatal death reviews, advanced as an important strategy to reduce stillbirth in sub-Saharan Africa settings [30]. In HIC settings, organisations are increasingly encouraged to move away from a focus on blaming individuals, towards acknowledging systems factors, alongside individual responsibility, and learning [31]. This ‘Just’ or ‘Responsibility’ culture has advantages in incorporating support for staff, even when mistakes have been made. There is a lack of evidence to surrounding specific interventions to provide psychological support for health workers after adverse events in maternity care in LMICs. Experience in HIC settings suggests that positive action, including prompt identification of needs after an incident, peer support through individual or group debriefing and referral for professional counselling were helpful [32]. These interventions are most likely to be successfully applied within a proactive management structure in facilities. Improving the workplace culture depends on effective leadership at all levels, as the changes required will involve considerable institutional commitment, policy and system level support, and this may be challenging where resources are stretched [33].

### Strengths and limitations

Although respectful and compassionate bereavement care is recognised as a key influence on adjustment and recovery after the death of a baby, the experiences of staff in maternity facilities has received scant attention [34], particularly in LMICs [8]. This study represents the most extensive exploration of the experiences of health workers providing care to women and families across urban and more rural facilities in Kenya and Uganda, sub-Saharan African countries with high burdens of stillbirth. Combining data gathered across multiple sites, in two countries, may have reduced visibility of country-specific variations in experiences. However, separate initial analyses identified considerable commonalities across the data and local differences have been highlighted where identified. Furthermore, discussions with our partner stakeholder and CEI groups across the NIHR Stillbirth Global Health Research Group in Malawi, Tanzania, Zambia and Zimbabwe indicated resonance of experiences across the network. However, the findings might not be transferable to other sub-Saharan Africa settings. As most births now occur in health facilities, midwives, nurses and doctors providing care in these settings were the focus. However, traditional birth attendants (TBAs) retain influence and status, particularly in remote and rural communities and their perspectives might also be helpful, as would those of community health workers.

## Conclusion and recommendations

Health workers in this study were highly cognisant of the impacts of stillbirth on women and families in Kenya and Uganda. Providing care after the death of a baby was arduous, emotionally challenging, and often unsatisfying due to the perceived shortcomings in quality. An overwhelming majority recognised the urgent need for better support for bereaved parents, in this key period immediately after the baby’s death. Enhancing knowledge and skills through context appropriate bereavement education would undoubtedly contribute to increasing health workers’ confidence. However, interventions should also target a shift in organisational culture which nurtures and sustains health workers in this role. Recommendations include, reorientating the prevailing culture from ‘blame’ to ‘learning and support’, providing opportunities for supervision and debriefing to share experiences are potential strategies which could also enhance health workers wellbeing and ability to care for bereaved families.

## Author contributions

The study was conceptualised, by TM and TL with the contribution of EA, GO and SW. EA, GO, SW, JM, AN, ANe, RM participated in data collection. Data was analysed by JM, AN, ANe, RM and TM. All authors contributed to interpretation of the data. TM drafted the manuscript with input from TL. All authors reviewed and approved the final manuscript.

## Ethical statement

The study was approved by the Research and Ethics Committees of; The University of Manchester UREC 2017-0233-4462; Makerere University School of Health Sciences SHSREC 2017-097: 27/04/2018 and University of Nairobi P240/05/2017: 23/06/2017. Ethical approval was also obtained from the Uganda National Council for Science and Technology SS 4666:12/07/2018. Administrative clearance was also sought from the respective hospitals. Written informed consent was obtained from all participants, including for use of anonymised verbatim quotes. All study processes including recruitment, data collection and data processing were carried out in accordance with relevant guidance and regulations.

## Funding

This study was funded by a Wellbeing of Women/RCM/ Burdett Trust International Fellowship (IFA 200) and by the National Institute for Health Research (NIHR; 16/137/53) using UK aid from the UK Government to support global health research. The views expressed in this publication are those of the author(s) and not necessarily those of the NIHR or the UK Department of Health and Social Care. The funding body had no role in the design, collection, analysis or interpretation of the data.

## Conflicts of interest

The authors declare they have no conflict of interests.

## Data Availability

The de-identified data sets used and/or analysed during the current study are not publicly archived but are available from the corresponding author on reasonable request.
